# Protective effect of HTK solution on postoperative pulmonary function in infants with CHD and PAH

**DOI:** 10.1042/BSR20170984

**Published:** 2017-12-07

**Authors:** Jindong Li, Yanhong Wu, Xudong Tian, Jiantang Wang, Mingfeng Dong, Anbiao Wang, Shengjun Ma

**Affiliations:** 1 Department of Cardiac Surgery, Liao Cheng People’s Hospital, Liaocheng 252000, Shandong, China; 2 Department of Cardiac Surgery, Shandong Provincial Hospital Affiliated to Shandong University, Jinan 250021, Shandong, China

**Keywords:** Congenital heart disease, HTK, Lung function, Pulmonary arterial hypertension, Pulmonary arterial perfusion

## Abstract

Objective: In the present study, we aimed to investigate the effect of pulmonary arterial perfusion (PAP) with Histidine–tryptophan–ketoglutarate (HTK) on lung protection in infants with congenital heart disease (CHD) and pulmonary arterial hypertension (PAH) after cardiopulmonary bypass (CPB).

Methods: Fifty infant patients with CHD and PAH at our hospital from January, 2016 to February, 2017 were randomly divided into control group and HTK group. The levels of interleukin-6 (IL-6), malondialdehyde (MDA), and endothelin-1 (ET-1) in serum were detected using ELISA Kit. Oxygen index (OI) and respiratory index (RI) were calculated at each time point. The time of postoperative mechanical ventilation and ICU stay was counted, and the right lower lung tissues in patients were taken for pathological examination.

Results: Compared with preanesthesia, the levels of IL-6, MDA, and ET-1 in the two groups were significantly increased after CPB, and their levels in HTK group were significantly lower than that in control group. Moreover, OI in control group decreased markedly and RI in control group increased significantly after CPB. Compared with control group, the postoperative mechanical ventilation time, postoperative ICU stay, and total hospital stay in HTK group were markedly short. In addition, inflammatory cells infiltration decreased and pulmonary interstitial showed mild edema in HTK group.

Conclusion: PAP with HTK could effectively reduce CPB-induced lung injury and improve lung function.

## Introduction

With the improvement of cardiopulmonary bypass (CPB), open heart surgery has a higher safety in the treatment of infant with congenital heart disease (CHD). However, postoperative pulmonary dysfunction is still a common clinical problem, especially for CHD with pulmonary arterial hypertension (PAH) in infants, which can often cause acute respiratory distress syndrome (ARDS) or even death. Therefore, how to alleviate lung injury after CPB is still a problem to be solved imperatively.

The mechanism of CPB-induced lung injury has not been fully elucidated, which is affected by many factors. At present, CPB-induced lung injury is mainly related to CPB-induced systemic inflammatory response syndrome (SIRS), lung ischemia/reperfusion (IR) injury, and respiratory mechanical ventilation [1,2]. Pulmonary arterial perfusion (PAP) with hypothermic protective solution (HPS) is one of the measures to reduce lung IR injury. In animal experiments and clinical applications, PAP of HPS has a significant curative effect to reduce the CPB-induced lung injury. Siepe et al. [3] found that PAP with hypothermic oxygenated blood during CPB had a good protective effect on lung function. Suzuki et al. [4] found that performed PAP with hypothermic oxygenated blood in the perfusion group could get higher PaO_2_/FiO_2_ and significantly reduce the duration of ventilation (DV) after operation, thus effectively prevented lung injury. Schlensak et al. [5] also demonstrated that PAP with hypothermic oxygenated blood could effectively reduce lung injury during CPB.

Histidine–tryptophan–ketoglutarate (HTK) solution was found by Bretschneider and co-workers, and initially used as a cardioplegic solution for cardiac surgery [6]. At present, HTK is considered as the ideal organ protection solution. Researches have confirmed that HTK had protective effects on hearts in warm ischemia. Li et al. [7] found that HTK had a better myocardial protective effect. However, there are few reports on HTK in lung protection. Luh et al. [8] demonstrated that HTK significantly reduced the content of myeloperoxidase (MPO) compared with Euro–Collins (EC) solution. Buggeskov et al. [9] found that PAP with hypothermic HTK could improve lung function.

In the present study, the levels of interleukin-6 (IL-6), malondialdehyde (MDA), and endothelin-1 (ET-1) in the serum and the change of oxygen index (OI) and respiratory index (RI) were measured to investigate the effect of PAP with HTK on lung protection in infant with CHD after CPB.

## Material and methods

### Patients

Fifty infant patients (28 male and 22 female) with CHD and PAH at our hospital from January, 2016 to February, 2017 were included in the study. The mean age was 12 ± 4.5 months (8–24 months), and the mean weight was 9.8 ± 3.4 kg (8.2–19.5 kg). It included 15 cases with ventricular septal defect (VSD), 13 cases with VSD and atrial septal defect (VSD + ASD), 9 cases with VSD and patent ductus arteriosus (VSD + PDA), and 5 cases with atrioventricular septal defect (AVSD), and 8 cases with total anomalous pulmonary venous drainage (TAPVD). The diagnosis was based on the clinical features, electrocardiogram (ECG), X-ray chest radiography, color Doppler echocardiography (CDE), and blood gas analysis. Patients were randomly divided into two groups: control group (25 cases) and perfusion group (HTK group, 25 cases, lung protection was carried out with HTK solution). The distribution of disease in each group was basically same. Patients all had a history of repeated respiratory tract infection before operation. X-ray chest radiography revealed pulmonary congestion, both ventricular enlargement and pulmonary artery elevation. The mean cardiothoracic ratio was 0.60 ± 0.15 (0.55–0.80). Pulmonary arterial systolic pressure was 55 ± 18 mmHg (50–80 mmHg, 1 mmHg = 0.133 kPa). The mean ratio of systolic pulmonary artery pressure to systemic circulation systolic pressure (Pp/Ps) was 0.74 ± 0.16 (0.60–0.85).

The study protocol was approved by the Medical Ethics Committee of Liao Cheng People’s Hospital. The records of all patients were analyzed retrospectively.

### Operation methods

All patients were performed with tracheal intubation anesthesia, underwent median sternotomy, and opened pericardium to reveal the heart. CPB was established though an arterial cannula in the aortic root (AR), and venous drainage tube was inserted into superior and inferior vena cava. In perfusion group, a perfusion tube was inserted into the root of main pulmonary artery (MPA) simultaneously. For myocardial protection, the two groups were perfused 4°C HTK (40 ml/kg) via aortic root. The perfusion time was 7 min. The perfusion group was simultaneously perfused with 4°C HTK (25 ml/kg) via MPA, and perfusion pressure was 0–40 cm H_2_O. In control group, the pulmonary artery was not perfused with HTK. After HTK perfusion, correction of cardiac malformation was performed. The composition of HTK include: 15 mmol/l NaCl, 9 mmol/l KCl, 4 mmol/l MgCl, 18 mmol/l histidine hydrochloride, 30 mmol/l mannitol, 1.0 mmol/l α-ketoglutarate, 2 mmol/l histidine, and 180 mmol/l tryptophan. pH value is 7.3 and osmotic pressure is 310 mOsmol/l.

### Testing index

The arterial blood (2 ml) was taken from all patients at preanesthesia, 0, 6, 12, 24, 48 h after CPB. The levels of IL-6, MDA, and ET-1 in serum were detected using ELISA Kit (Shanghai Enzyme-linked Biotechnology Co., Ltd., China) following the manufacturer’s instructions. Blood gas analysis was measured by blood gas analyzer (ABLTM77, Radiometer Medical ApS, Denmark), and OI and RI were calculated at each time point. Then, the time of postoperative mechanical ventilation and ICU stay in two groups were counted, and the right lower lung tissues in patients were taken for pathological examination after agreement of the patients’ family members.

$$\text{OI}={\text{PaO}}_{2}/{\text{FiO}}_{2}$$

$$\text{RI}=\text{P}(\text{A}-\text{a}){\text{O}}_{2}/{\text{PaO}}_{2}$$

$$\text{P}(\text{A}-\text{a}){\text{O}}_{2}=(\text{Pa }-\;{\text{PH}}_{2}\text{O})\;\times \;{\text{FiO}}_{2\;}-\;{\text{PaCO}}_{2}/\text{R}\;-\;{\text{PaO}}_{2}$$

PaO_2_: arterial partial pressure of oxygen; PaCO_2_: arterial partial pressure of carbon dioxide; FiO_2_: fraction of inspired oxygen; P(A–a)O_2_: alveolar–arterial oxygen gradient.

Pa (atmospheric pressure) = 760 mmHg, PH_2_O (saturation water vapor pressure) = 47 mmHg, *R* = 0.8.

### Statistical analysis

SPSS13.0 software was used for statistical analysis. Student’s *t* test was performed to test the differences between the two groups. All data were presented as mean ± standard deviation (SD). *P*<0.05 was considered as statistically significant.

## Results

### The levels of IL-6, MDA, and ET-1 in serum

**Figure 1:**
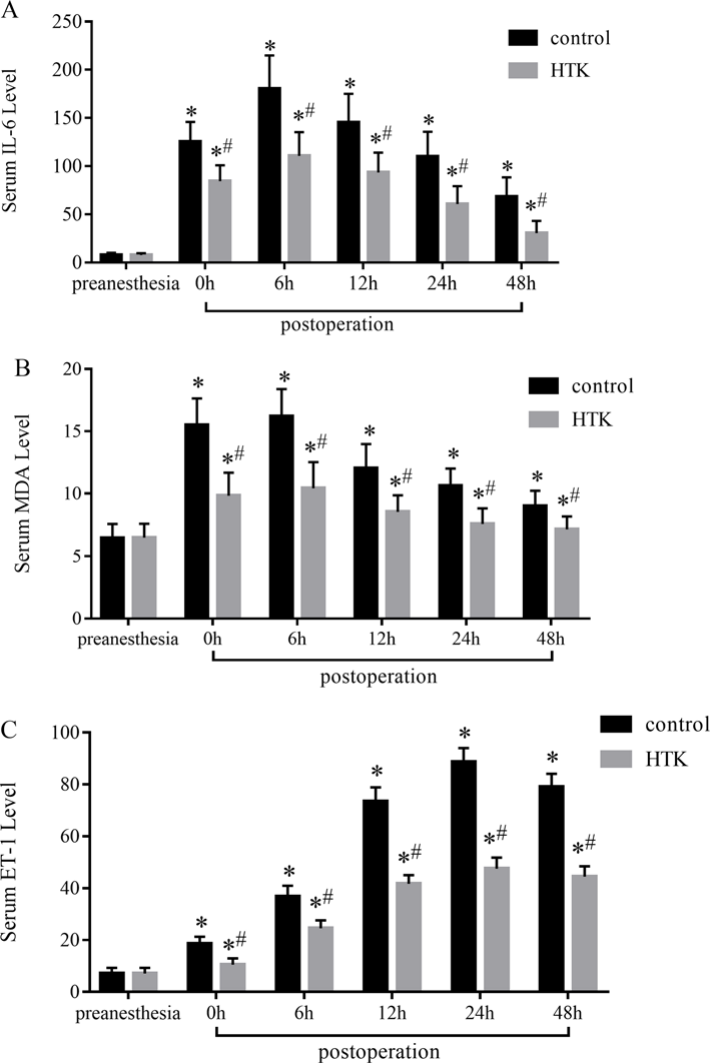
The level of IL-6, MDA and ET-1 in serum (**A**) IL-6 level, (**B**) MDA level, and (**C**) ET-1 level. The levels of IL-6, MDA, and ET-1 in the two groups were also significantly increased after CPB. The levels of IL-6 and MDA in serum peaked at 6 h after CPB, and then decreased gradually. The level of ET-1 reached the highest at 24 h after CPB. Moreover, the levels of IL-6, MDA, and ET-1 in HTK group were lower than that in control group at each time point after CPB. ^*^P<0.01, vs preanesthesia; ^#^*P*<0.05, vs control group.

As shown in Figure 1, the levels of IL-6 (Figure 1A), MDA (Figure 1B), and ET-1 (Figure 1C) in HTK group and control group had no significant difference at preanesthesia, but were significantly increased after CPB (*P*<0.01). The levels of IL-6 and MDA in serum peaked at 6 h after CPB, and then decreased gradually. The levels of ET-1 reached the highest at 24 h after CPB. Moreover, the levels of IL-6, MDA, and ET-1 in HTK group were lower than that in control group (*P*<0.05) at each time point after CPB.

### Lung function index

**Figure 2:**
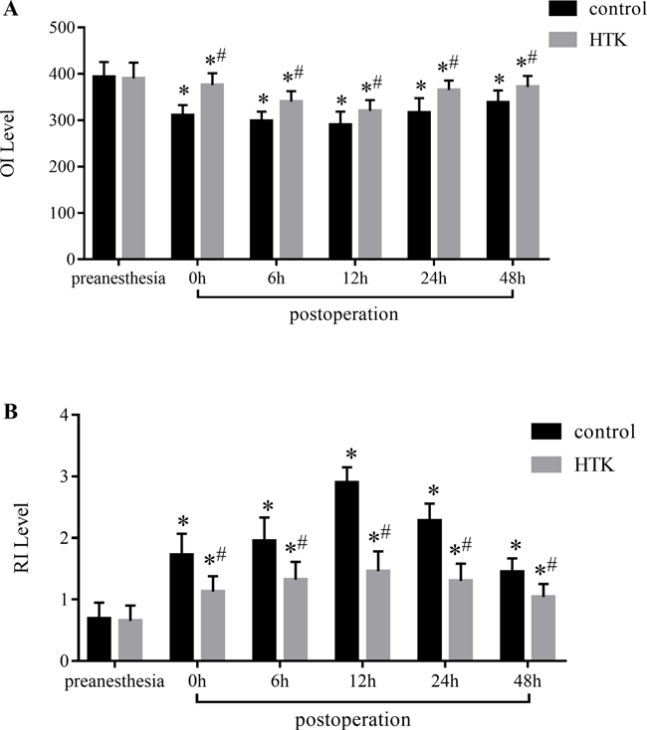
Lung function index (**A**) OI level and (**B**) RI level. OI is the most commonly used index in clinic, which can directly reflect the degree of respiratory failure and the effect of ventilation in children. RI is an index to evaluate the decrease in respiratory function, which can accurately reflect the ventilation of children. ^*^P<0.01, vs preanesthesia; ^#^*P*<0.05, vs control group.

OI is the most commonly used index in clinic, which can directly reflect the degree of respiratory failure and the effect of ventilation in children. RI is an index to evaluate the decrease in respiratory function, which can accurately reflect the ventilation of children. At preanesthesia, OI (Figure 2A) and RI (Figure 2B) in HTK group and control group had no significant difference, but OI in control group decreased markedly (*P*<0.01) and RI in control group increased significantly after CPB (*P*<0.01).

### Clinical index

**Figure 3:**
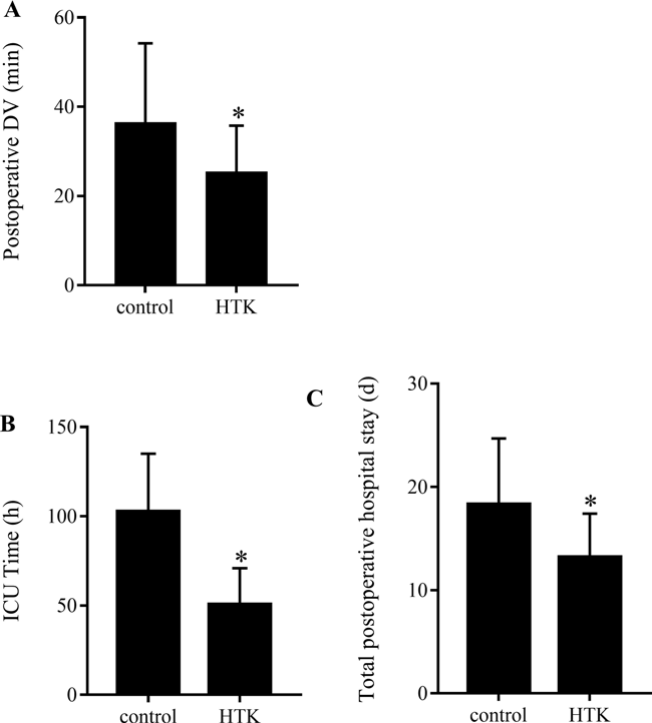
Clinical index (**A**) The postoperative duration of ventilation (DV), (**B**) postoperative ICU stay, and (**C**) total hospital stay. The postoperative duration of DV in HTK group was shorter than in control group. Postoperative ICU stay and total hospital stay in HTK group were also significantly less than those in control group; *P<0.05, vs control group.

All the 50 patients were cured and no death. Compared with control group, the postoperative mechanical ventilation time of HTK group was significantly short (Figure 3A). In addition, postoperative ICU stay (Figure 3B) and total hospital stay (Figure 3C) in HTK group were significantly less than those in control group.

### Pathological results of lung tissue

**Figure 4:**
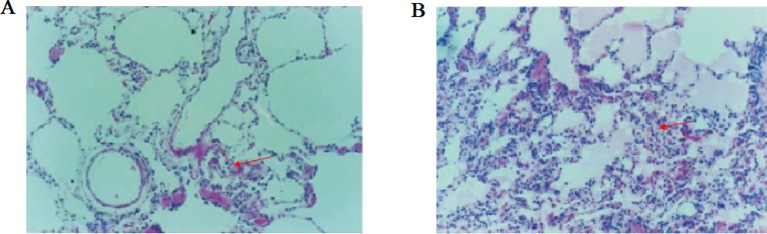
Pathological histology analysis results (**A**) HTK group and (**B**) control group. The black arrow is point to neutrophils. Compared with HTK group, a large number of neutrophils were seen in control group, and the edema of alveoli and pulmonary interstitial was obvious.

The pathological results showed that neutrophils infiltration in lung tissues decreased, and pulmonary interstitial had mild edema in HTK group (Figure 4A). However, in control group (Figure 4B), a large number of neutrophils infiltrated in lung tissues, and the edema of alveoli and pulmonary interstitial was obvious.

## Discussion

Lung function injury is one of the most common complications after CPB. Particularly, there are obvious pulmonary vascular lesion before surgery, such as vascular endothelial injury, the edema of alveoli and pulmonary interstitial and atelectasis were common in the patients with severe PAH after CPB, which severely affects gas exchange and hemodynamic stability in lung. It was reported that the incidence of acute lung injury (ALI) after CPB in pediatric patients with PAH was significantly higher than that in adults, and this complication could lead to high mortality [10]. Therefore, the active prevention and treatment of lung injury in CPB can help to reduce postoperative pulmonary complications and improve the success rate of operation.

HTK is an ideal preservation solution for organ. Lee et al. [11] found that the myocardium preserved in HTK had good cardiac function recovery. Dolińska et al. [12] reported that HTK and Biolasol as kidney preservation solutions could improve graft function and survival rate. Becker et al. [13] found that HTK and UW solution had a good protective effect on the ischemic pancreas. In the present study, we incorporated the PAP method with the organ protective effects of HTK during CPB to explore an effective method for lung protection.

The results of the present study showed that the level of IL-6 in the serum, an identification factor of the extent of acute inflammation in endothelial cells, was significantly increased in the two groups after CPB. And the level of IL-6 in HTK group was significantly lower than that in control group. This suggested that PAP with HTK could decrease the release of inflammatory mediators and reduce the inflammatory response of lung after CPB. Its mechanism might be closely related to its composition: (1) HTK contains α-ketoglutarate and tryptophan. α-Ketoglutarate is the intermediate product of tricarboxylic acid cycle, which can produce ATP by the tricarboxylic acid cycle, respiratory chain, and oxidative phosphorylation [14]. Akbar et al. [15] confirmed that HTK increased the level of cyclic adenosine monophosphate (cAMP) during ischemia, which was beneficial to the preservation of cell integrity. (2) The low K^+^ and histidine buffer in HTK can maintain the stability of ATP level during ischemia [16], protect the vascular endothelial cells, and reduce the release of inflammatory factors by endothelial cells. Cunningham et al. [17] confirmed that HTK had a good protective effect on the function of endothelial cells.

Furthermore, ischemia/reperfusion (I/R) tissues can produce a large number of oxygen free radicals (OFR), and these OFR react with polyunsaturated fatty acids, which can produce a large number of toxic lipid peroxidation products, including MDA (the most toxic). Therefore, the detection of MDA content in serum can reflect the rate and extent of peroxidation *in vivo*. In the present study, we found that the levels of MDA in the two groups after CPB increased significantly compared with preoperative, but the levels of MDA in HTK group were markedly lower than those in control group, which confirmed that PAP with HTK could reduce the generation of OFR and lung I/R injury after CPB. The mechanisms might include: (1) HTK contains Mg^2+^, which plays an important role in maintaining the integrity of the cell membrane [18]. Mg^2+^ can reduce Ca^2+^ influx by competing ion channel with Ca^2+^, and then mitigate calcium overload and the generation of OFR. In addition, Mg^2+^ is an activator of various ATP enzymes, but is easily lost during ischemia. HTK can effectively improve metabolic recovery after ischemia by supplementing Mg^2+^ [19]. (2) HTK contains mannitol, which not only has the effect of increasing osmotic pressure and dehydration, but also has strong OFR scavenging ability.

ET-1 is a potent vasoconstrictor released by endothelial cells. Thus, the level of ET-1 in serum can be used as a diagnostic index of endothelial cells injury [20]. In the present study, we found that the levels of ET-1 in the two groups after CPB were significantly increased compared with preoperative, but the levels in HTK group were lower than that in control group, which suggested that PAP with HTK can reduce the ET-1 generation and the injury of lung endothelial cells after CPB.

OI can reflect the human tissues oxygenation status, and is related to intrapulmonary shunt. The results of our study showed that OI was significantly better in HTK group than in control group, suggesting that HTK could improve the oxygenation capacity of lung. RI can accurately reflect the lung ventilation function, and is not affected by intracardiac shunt. Therefore, it is a good index for judging oxygen carrying capacity of the blood from the alveoli. In the present study, RI in HTK group was markedly better than in control group, which confirmed that HTK improved lung respiratory function.

In summary, the present study confirmed that PAP with HTK could effectively reduce CPB-induced lung injury and then improve lung function, which would provide a new approach to reduce postoperative pulmonary complications in infants with CHD and PAH.

## Abbreviations

ALI: acute lung injury
AR: aortic root
ARDS: acute respiratory distress syndrome
CDE: color Doppler echocardiography
CHD: congenital heart disease
CPB: cardiopulmonary bypass
DV: duration of ventilation
EC: Euro–Collins
ECG: electrocardiogram
HTK: histidine–tryptophan–ketoglutarate
HPS: hypothermic protective solution
IR: ischemia/reperfusion
MPA: main pulmonary artery
MPO: myeloperoxidase
OFR: oxygen free radicals
OI: oxygen index
PAH: pulmonary arterial hypertension
PAP: pulmonary arterial perfusion
RI: respiratory index
SIRS: systemic inflammatory response syndrome
TAPVD: total anomalous pulmonary venous drainage
VSD: ventricular septal defect

## References

[1] Xing Z., Han J., Hao X., Wang J., Jiang C., Hao Y. (2017) Immature monocytes contribute to cardiopulmonary bypass-induced acute lung injury by generating inflammatory descendants. Thorax 72, 245 27660037 10.1136/thoraxjnl-2015-208023

[2] Romano R., Tatham K., O’Dea K., Sarathchandra P., Thakuria L., Reed A. (2015) OP-038 – Impact of cardiopulmonary bypass (CPB) on lung injury and marginated leukocyte dynamics during bilateral lung transplantation (LTx). J. Cardiothorac. Vasc. Anesth. 29, S38–S39

[3] Siepe M., Goebel U., Mecklenburg A., Doenst T., Benk C., Stein P. (2008) Pulsatile pulmonary perfusion during cardiopulmonary bypass reduces the pulmonary inflammatory response. Ann. Thorac. Surg. 86, 115 18573409 10.1016/j.athoracsur.2008.03.062

[4] Suzuki T., Fukuda T., Ito T., Inoue Y., Cho Y. and Kashima I. (2000) Continuous pulmonary perfusion during cardiopulmonary bypass prevents lung injury in infants. Ann. Thorac. Surg. 69, 602–606 10735706 10.1016/s0003-4975(99)01332-6

[5] Schlensak C., Doenst T., Preusser S., Wunderlich M., Kleinschmidt M. and Beyersdorf F. (2002) Cardiopulmonary bypass reduction of bronchial blood flow: a potential mechanism for lung injury in a neonatal pig model. J. Thorac. Cardiovasc. Surg. 123, 1199–1205 12063469 10.1067/mtc.2002.121977

[6] Hölscher M. and Groenewoud AF. (1991) Current status of the HTK solution of Bretschneider in organ preservation. Transplant. Proc. 23, 2334–7 1926380

[7] Li S., Wu J., Watanabe M., Li C. and Okada T. (2006) Protective effects of ischemic postconditioning against hypoxia-reoxygenation injury and hydrogen peroxide-induced damage in isolated rat hearts. Exp. Clin. Cardiol. 11, 280–285 18651018 PMC2274845

[8] Luh S.P., Yang P.C., Lee C.J., Tsai T.P. and Wang Y.H. (2004) Comparison of histidine-tryptophan-ketoglutarate and Euro-Collins solutions for lung preservation using the minipig in situ warm ischemia model. J. Formos. Med. Assoc. 103, 292–296 15175825

[9] Buggeskov K.B., Wetterslev J., Secher N.H., Andersen L.W., Jonassen T. and Steinbruchel D.A. (2013) Pulmonary perfusion with oxygenated blood or custodiol HTK solution during cardiac surgery for postoperative pulmonary function in COPD patients: a trial protocol for the randomized, clinical, parallel group, assessor and data analyst blinded Pulmonary Protection Trial. Trials 14, 30 23363494 10.1186/1745-6215-14-30 PMC3576307

[10] Shi S.S., Chen C., Zhao D.Y., Liu X.W., Cheng B.L., Wu S.J. (2014) The role of plasma gelsolin in cardiopulmonary bypass induced acute lung injury in infants and young children: a pilot study. BMC Anesthesiol. 14, 67 25126004 10.1186/1471-2253-14-67 PMC4132929

[11] Lee S., Huang C.S., Kawamura T., Shigemura N., Stolz D.B., Billiar T.R. (2010) Superior myocardial preservation with HTK solution over Celsior in rat hearts with prolonged cold ischemia. Surgery 148, 463–473 20627336 10.1016/j.surg.2010.04.009

[12] Dolińska B., Ostróżka-Cieślik A., Caban A., Cierpka L. and Ryszka F. (2012) Comparing the effect of Biolasol® and HTK solutions on maintaining proper homeostasis, indicating the kidney storage efficiency prior to transplantation. Ann. Transplant. 17, 74–78 22743725 10.12659/aot.883225

[13] Becker T., Ringe B., Nyibata M., Meyer zu V.A., Schrem H., Lück R. (2007) Pancreas transplantation with histidine-tryptophan-ketoglutarate (HTK) solution and University of Wisconsin (UW) solution: is there a difference? J. Pancreas 8, 304 17495359

[14] Korun O., Özkan M., Ay F.X., Terzie A.G. (2013) The comparison of the effects of Bretschneider’s histidine-tryptophan-ketoglutarate and conventional crystalloid cardioplegia on pediatric myocardium at tissue level. Artif. Organs 37, 76–81 23305576 10.1111/j.1525-1594.2012.01575.x

[15] Akbar S. and Minor T. (2001) Significance and molecular targets of protein kinase A during cAMP-mediated protection of cold stored liver grafts. Cell. Mol. Life Sci. 58, 1708 11706996 10.1007/PL00000808 PMC11337289

[16] Edelman J.J., Seco M., Dunne B., Matzelle S.J., Murphy M., Joshi P. (2013) Custodiol for myocardial protection and preservation: a systematic review. Ann. Cardiothorac. Surg. 2, 717–728 24349972 10.3978/j.issn.2225-319X.2013.11.10 PMC3857005

[17] Cunningham A., Wilson C., Stansby G. and Haswell M. (2004) Evaluation of eight different preservation solutions for endothelial in situ preservation. Commun. Part. Diff. Eq. 38, 304–338 10.1097/01.tp.0000135465.00738.ed 15480166

[18] Yokoyama K., Kimura M., Aoki H., Futami T. and Itoman M. (2010) Comparative study between Euro-Collins solution and UW solution for cold storage of rat limbs. Orthop. Traumatol. 44, 732–734

[19] Kim J.T., Park Y.H., Chang Y.E., Byon H.J., Kim H.S., Kim C.S. (2011) The effect of cardioplegic solution-induced sodium concentration fluctuation on postoperative seizure in pediatric cardiac patients. Ann. Thorac. Surg. 91, 1943–1948 21511247 10.1016/j.athoracsur.2011.02.003

[20] Comellas A.P. and Briva A. (2009) Role of endothelin-1 in acute lung injury. Transl. Res. 153, 263–271 19446279 10.1016/j.trsl.2009.02.007 PMC3046772

