# Correction: Protective effect of HTK solution on postoperative pulmonary function in infants with CHD and PAH

**DOI:** 10.1042/BSR20170984_COR

**Published:** 2018-11-30

**Authors:** Jindong Li, Yanhong Wu, Xudong Tian, Jiantang Wang, Mingfeng Dong, Anbiao Wang, Shengjun Ma

***Bioscience Reports* (2017) vol. 37, BSR20170984; https://doi.org/10.1042/BSR20170984**

The author Shengjun Ma was omitted from the Author Contribution section of the published version of this article. S.M. reviewed the manuscript and put forward valuable suggestions for revision.

